# Characterization of the complete mitochondrial genome of *Oryzaephilus surinamensis* Linne (Insecta: Coleoptera: Silvanidae) from Xichuan

**DOI:** 10.1080/23802359.2019.1692714

**Published:** 2019-11-20

**Authors:** Sheng Liang, Yu Bai, Jun Chen, Bocheng Ouyang

**Affiliations:** a College of Mathematics & Information Science, Guiyang University, Guiyang, Guizhou, China;; b Guizhou Provincial Key Laboratory for Rare Animal & Economic Insects of the Mountainous Region, Guiyang University, Guiyang, Guizhou, China;; c School of Electronic & Communication Engineering, Guiyang University, Guiyang, Guizhou, China

**Keywords:** *Oryzaephilus surinamensis*, Silvanidae, saw-toothed grain beetle, mitochondrial genome, stored-product insect

## Abstract

The saw-toothed grain beetle, *Oryzaephilus surinamensis* Linné, is a well-known stored-product insect. Beetles were obtained from Xichuan County and the mitochondrial genome was characterized (GenBank accession number MN535903). The mitogenome consists of a circular DNA molecule of 15,941 bp, with only 27.36% GC content. It comprises 13 protein-coding, 22 tRNA, and 2 rDNA genes. The protein-coding genes have typical ATN (Met) initiation codons and are terminated by typical TAN stop codons.

The saw-toothed grain beetle, *Oryzaephilus surinamensis* Linné, is a stored-product insect found in vegetable foods worldwide (Back and Cotton 1926). Although this organism has been known to scientists for more than 240 years (Back and Cotton 1926), its mitochondrial genome has not been sequenced completely. Here, we have characterized the complete mitogenome of *O. surinamensis* to better comprehend its molecular evolution and taxonomic classification.

Samples of adult *O. surinamensis* (Specimen Accession Number: GYU-20190630-001) were obtained from Jingziguan town (E 111.026°, N 33.244°), Xichuan County, Nanyang City, Henan Province, China, on 30 June 2019. Genomic DNA was isolated and fragmented to build a genomic library of insert Size 400 bp that was sequenced (paired end 2 × 150 bp) using an Illumina HiSeq 4000 (San Diego, CA). We obtained 24,372,898 reads of raw data, 24,012,730 of which were high-quality, clean data (98.52%). The genome was assembled de novo with A5-miseq v20150522 (https://github.com/koadman/docker-A5-miseq) (Coil et al. 2014) and SPAdes v3.9.0 (http://cab.spbu.ru/software/spades/) (Bankevich et al. 2012).

The mitogenome of *O. surinamensis* consists of a 15,941 bp circular DNA molecule with 31.68% A, 40.96% T, 9.2% C, and 18.17% G, which is an A/T bias of 72.64%. The AT- and GC-skews of the major strands of the mitogenome were calculated to be approximately −0.12775 and 0.32773, respectively. The A/T-rich region in the mitogenome is 1455 bp, with a 75.26% A + T content, and is located between the srRNA and tRNA-Ile.

The mitogenome of *O. surinamensis* contains 13 protein-coding genes (PCGs), 22 tRNA genes, and 2 rRNA genes. The order and orientation of the *O. surinamensis* mitogenome functional areas are identical to those of *Tenebrio obscurus*, *Zophobas atratus*, and *Blaps rynchopetera* mitogenome functional areas (Bai et al. 2018; Bai et al. 2019; Yang et al. 2019). All 13 PCGs have typical ATN (Met) start codons and TAN stop codons. The other genes have alternative start codons: *nad1* and *atp6* – ATA; *nad5*, *nad3*, *atp8*, *nad6*, and *cox2* – ATT; *cox3*, *nad4*, *cob*, and *nad4l* – ATG; *nad2* and *cox1* – ATC. Three genes (*nad4l*, *atp6*, and *nad6*) have a TAA stop codon; three genes (*nad1*, *cob*, and *nad5*) have a TAG stop codon, and seven genes (*nad4*, *nad3*, *cox3*, *atp8*, *cox2*, *cox1*, and *nad2*) have an incomplete stop codon consisting of a T that is completed by the addition of 3′ A nucleotides to the resultant mRNA. The 22 tRNA genes are interspersed throughout the coding region and range from 58 (tRNA-Ser) to 70 bp (tRNA-Lys). The lrRNA and srRNA genes in the *O. surinamensis* mitogenome are 1247 and 757 bp long.

To validate the phylogenetic position of *O. surinamensis*, the mitogenome DNA sequences from 16 species of Cucujiformia were used to construct a phylogenetic tree by the maximum-likelihood method using the MEGA 7 software (Kumar et al. 2016) (Figure 1). Overall, our study provides insight into the mitogenome of *O. surinamensis*, which will be useful for its taxonomic classification and further phylogenetic reconstruction.

**Figure 1.**
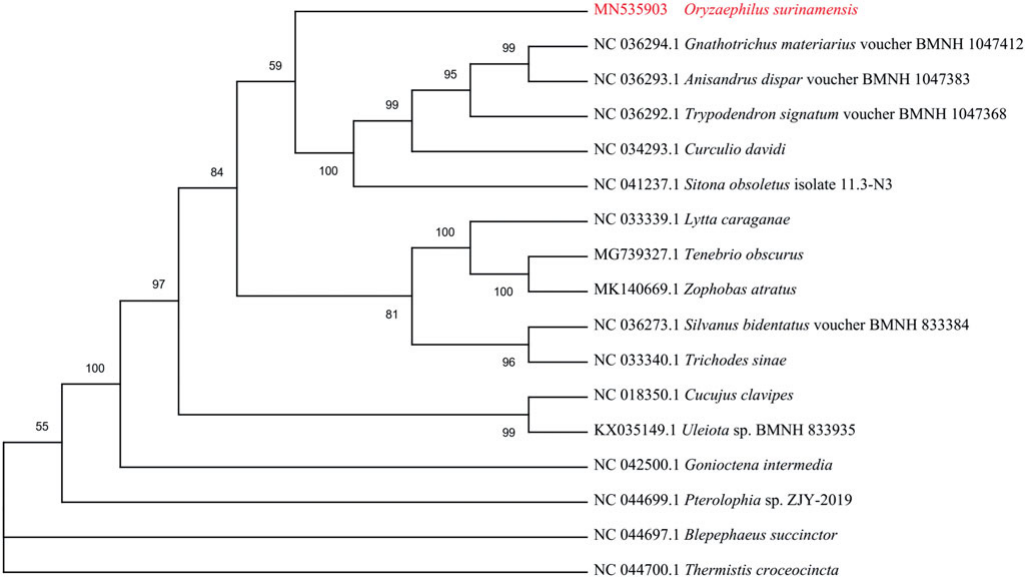
The maximum-likelihood phylogenetic tree of *O. surinamensis* and 16 other Cucujiformia beetles based on mitogenome DNA sequences.

## References

[CIT0001] Back E, Cotton R 1926 Biology of the saw-toothed grain beetle *Oryzaephilus surinamensis* Linne. J agric Res. 33:435–452.

[CIT0002] Bai Y, Li C, Yang M, Liang S 2018 Complete mitochondrial genome of the dark mealworm *Tenebrio obscurus* Fabricius (Insecta: Coleoptera: Tenebrionidae). Mitochondrial DNA Part B. 3(1):171–172. 10.1080/23802359.2018.1437800 PMC7799911 33474107

[CIT0003] Bai Y, Wang H, Li G, Luo J, Liang S, Li C 2019 Complete mitochondrial genome of the super mealworm *Zophobas atratus* (Fab.) (Insecta: Coleoptera: Tenebrionidae). Mitochondrial DNA Part B. 4(1):1300–1301.

[CIT0004] Bankevich A, Nurk S, Antipov D, Gurevich AA, Dvorkin M, Kulikov AS, Lesin VM, Nikolenko SI, Pham S, Prjibelski A 2012 SPAdes: a new genome assembly algorithm and its applications to single-cell sequencing. J Comp Biol. 19(5):455–477. 10.1089/cmb.2012.0021 PMC3342519 22506599

[CIT0005] Coil D, Jospin G, Darling A 2014 A5-miseq: an updated pipeline to assemble microbial genomes from Illumina MiSeq data. Bioinformatics. 31(4):587–589. 25338718 10.1093/bioinformatics/btu661

[CIT0006] Kumar S, Stecher G, Tamura K 2016 MEGA7: molecular evolutionary genetics analysis version 7.0 for bigger datasets. Mol Biol Evol. 33(7):1870–1874. 27004904 10.1093/molbev/msw054 PMC8210823

[CIT0007] Yang Y, Bai Y, Zheng J, Chen J, Ouyang B, Liang S 2019 Characterization of the complete mitochondrial genome of *Blaps rynchopetera* Fairmaire (Insecta: Coleoptera: Tenebrionidae) from Dali. Mitochondrial DNA Part B. 4(2):3167–3168. 10.1080/23802359.2019.1667905 PMC7706892 33365902

